# Genome-Guided Phylo-Transcriptomic Methods and the Nuclear Phylogenetic Tree of the Paniceae Grasses

**DOI:** 10.1038/s41598-017-13236-z

**Published:** 2017-10-19

**Authors:** Jacob D. Washburn, James C. Schnable, Gavin C. Conant, Thomas P. Brutnell, Ying Shao, Yang Zhang, Martha Ludwig, Gerrit Davidse, J. Chris Pires

**Affiliations:** 1 Division of Biological Sciences, 311 Bond Life Sciences Center, University of Missouri, Columbia, MO 65211 USA; 2 Department of Agronomy & Horticulture, Beadle Center E207, University of Nebraska-Lincoln, Lincoln, NE 68588 USA; 3 Donald Danforth Plant Sciences Center, 975N Warson Rd., St. Louis, MO 63132 USA; 4 Division of Animal Sciences, 920 East Campus Drive, University of Missouri, Columbia, 65211 MO USA; 5 Program in Genetics, Bioinformatics Research Center, Department of Biological Sciences, 356 Ricks Hall, North Carolina State University, Raleigh, NC 27695 USA; 6 St. Jude Children´s Research Hospital, MS 342, Room D-4047E, 262 Danny Thomas Place, Memphis, TN 38105 USA; 7 School of Molecular Sciences, The University of Western Australia (M310), 35 Stirling Highway, Crawley, WA 6009 Australia; 8 Missouri Botanical Garden, P.O. Box 299, St. Louis, Missouri 63166-0299 USA

## Abstract

The past few years have witnessed a paradigm shift in molecular systematics from phylogenetic methods (using one or a few genes) to those that can be described as phylogenomics (phylogenetic inference with entire genomes). One approach that has recently emerged is phylo-transcriptomics (transcriptome-based phylogenetic inference). As in any phylogenetics experiment, accurate orthology inference is critical to phylo-transcriptomics. To date, most analyses have inferred orthology based either on pure sequence similarity or using gene-tree approaches. The use of conserved genome synteny in orthology detection has been relatively under-employed in phylogenetics, mainly due to the cost of sequencing genomes. While current trends focus on the quantity of genes included in an analysis, the use of synteny is likely to improve the quality of ortholog inference. In this study, we combine *de novo* transcriptome data and sequenced genomes from an economically important group of grass species, the tribe Paniceae, to make phylogenomic inferences. This method, which we call “genome-guided phylo-transcriptomics”, is compared to other recently published orthology inference pipelines, and benchmarked using a set of sequenced genomes from across the grasses. These comparisons provide a framework for future researchers to evaluate the costs and benefits of adding sequenced genomes to transcriptome data sets.

## Introduction

Phylogenetic methods have undergone enormous changes over the past few years as the costs of next generation sequencing have declined. Where researchers once spent considerable time designing and testing PCR primers to sequence one or a few genes, it is now becoming common to sequence large numbers of genes, or even whole genomes, for phylogenomic analyses^1–9^. In an increasing number of cases, it is possible to build phylogenetic trees based on sequenced genomes, but even these are often re-sequenced or low coverage genomes^1,10–20^. For most groups of eukaryotic organisms, the costs of sequencing and assembling whole genomes remain prohibitive, limiting the applicability of whole genome sequencing for studies that sample large numbers of taxa. Whole genomes are also not generally necessary to allow phylogenomic methods to provide increased resolution of species relationships^21^. Reduced representation approaches, where part of the genome is excluded from sequencing, allow researchers to obtain sequence data for large numbers of nuclear genes across many species at a relatively low cost and have become increasingly common^22–29^.

The current study focuses on improving and testing the constraints of one of these approaches, transcriptome-based phylogenomics. Variations of this method have been applied to a range of organisms and scientific questions^6,8,30–45^. Transcriptome-based methods differ from other reduced representation approaches in the nature of the gene/transcript ascertainment bias that results. Transcriptomes produce a sampling of transcripts that are biased due to the biology of the organisms under study and the time point(s) and tissue(s) being sampled. Probe-based reduced representation methods on the other hand are biased by the methods used for discovering and choosing the probes. Transcriptome-based approaches to phylogenomics rely on sequencing RNA from multiple taxa at sufficient depth to enable *de novo* assembly of many (usually hundreds to thousands) of transcripts. The resulting transcripts are then used in phylogenetic analyses. The cost of sequencing transcriptomes is, of course, substantially less than that required for whole genomes. Transcriptome-based approaches also require less upfront time investment and a priori knowledge than probe hybridization/sequence capture-based methods. However, they require more bioinformatics time post-sequencing, the reason being that no probe design is required for transcriptome sequencing, but post-sequencing assembly is required. One key limitation of transcriptome-based methods is that they require access to fresh tissue or RNA, and therefore cannot be employed with, for example, museum collections. Conversely, one advantage of transcriptome-based methods is that the expression data can be used for additional biological analyses beyond phylogenetic inference, as RNA-Seq data is widely used to understand the evolution of gene expression^46–49^; of course, probe and hybridization-based methods can also be used for other types of functional exploration. Collecting and preserving RNA from fresh or frozen tissue has become routine in many laboratories^50^ and, at least in our hands, it is actually easier and less time consuming than DNA sequencing due to streamlined commercial kits (cited in materials and methods below) and the small quantities of RNA required for library preparation.

One area of rapid advancement in transcriptome-based phylogenomics (and most other phylogenomics approaches) is orthology determination. Once transcriptomes are generated and assembled, it is necessary to identify orthologous genes between the various transcriptomes; that is genes that are descended from a single gene copy present in the most recent common ancestor of the species being compared. To date, the most commonly used methods for orthology inference use sequence similarity-based methods like BLAST, often combined with the Markov Cluster (MCL) or other algorithms for differentiating between orthologs and paralogs. These platforms include OrthoMCL, HaMStR, OrthoFinder, and others^51–55^. While these methods have been useful for orthology inference, it is well established that sequence similarity can be misleading, and does not necessarily equate with orthology^51,56,57^. Some recent improvements on these methods start with all-by-all BLAST and/or MCL, but additionally use the topological features of phylogenetic trees to differentiate between orthologs and paralogs^52,58–60^. Two of the most advanced platforms for doing this are the Agalma and Yang & Smith pipelines^2,3,61–63^. These phylogenetically-informed methods have proven effective and become popular in large part because they are computationally tractable and because they require no *a priori* information about gene order (e.g., they do not require sequenced genomes). One of the major downsides to these methods is the use of an all-by-all BLAST step. Not only can sequence similarity searches be problematic for establishing orthology^51^, but when they are performed in an all-by-all framework they become extremely resource-intensive computationally. Some of these issues can be overcome through the phylogenetically informed approaches described above and the use of parallel computing, but many improvements remain to be made.

An alternative method for orthology inference that has been used in prokaryotes but received relatively little attention in eukaryotic phylogenetics is the use of gene synteny^64–69^. Synteny can be defined as the co-localization of the same gene at similar chromosomal positions across related taxa^66,70^. It has been compared to a street address system where, if one knows the physical location of a building, it is much easier to find that building than just looking for a building with specific features. Synteny-based orthology determination is then rooted in the assumption that orthologous genes will not only share sequence similarity, but will also reside in similar locations within the genomes of related species^70^. Synteny-based methods are widely employed in comparative genomics studies^69,71–77^. The omission of synteny-based approaches in most phylogenetic studies is likely due to the fact that syntenic analysis requires information on gene order in addition to gene sequence, and information on gene order is not captured by reduced representation methods, including amplification-based, probe-based and transcriptome-based datasets. Additionally, syntenic conservation levels vary depending on the phylogenetic proximity of the species being compared. However, for many phylogenetic clades, synteny is strongly conserved^70,78,79^, making it is possible to use syntenic data from a few genomes as an anchor for reduced representation data, an idea that has not yet been fully explored.

Here we describe the development and implementation of a method we call genome-guided phylo-transcriptomics. This method uses genome-derived syntenic orthologs to anchor transcripts for phylogenetic inference, and is here tested and applied in an economically and scientifically important group of grasses, the tribe Paniceae^80–85^. While the method still requires a BLAST step in which transcripts are mapped directly to reference genes that are known to be single-copy orthologs based on synteny, it bypasses the time consuming and error prone all-by-all BLAST and MCL algorithm steps commonly used in current phylo-transcriptomic methods^51,56,57^. Furthermore, by removing transcripts that map in multiple copies to the reference ortholog (see Materials and Methods section), one can avoid using BLAST to distinguish between paralogs and orthologs whose sequences are very similar. These are, of course, the sequences for which BLAST is most problematic^51^. We hypothesize that the use of a genome-guided method for orthology prediction will result in a greater percentage of “true” orthologs than those predicted by topology-based methods. This decrease in the signal-to-noise ratio in a data set could have serious impacts given the influence that even a single informative ortholog can have on a phylogenetic analysis^86^.

In addition to the Paniceae data set here generated, which covers 25–40 million years (m.y.) of evolutionary time, we applied the new method to a published dataset from the grape family (Vitaceae) to *Arabidopsis thaliana* which diverged 69–150 m.y. ago^2,81,82,87,88^. We also constructed and analyzed a data set from several publically available genomes from across the grasses (family Poaceae) and used it to benchmark the method’s reliability as compared to orthology inference based entirely on sequenced genomes. The three data sets were analyzed using both this genome-guided method as well as two recently published topology-based approaches for orthology inference with transcriptomes, the Agalma and Yang & Smith pipelines^2,3,62^.

## Materials and Methods

### Taxon Sampling and Plant Materials

Forty-five species from across the tribe Paniceae and outgroups were selected for RNA sequencing. Samples were obtained from the sources listed in Supplementary Table S1, with the majority of samples drawn from the USDA germplasm collection. Most samples were taken from the same plants as those used by Washburn *et al*.^80^ so results could be directly compared to the chloroplast phylogeny inferred in that study. Plants were grown and sampled in the greenhouse facilities at the University of Missouri, Columbia, MO and the Danforth Center, St. Louis, MO, with the exception of *Neurachne alopecuroidea* and *Paraneurachne muelleri*, for which RNA samples were obtained from Martha Ludwig, University of Western Australia. Leaf material was sampled from all plants and where possible, shoot, flower, and drought-stressed tissue samples were also taken with the hope of capturing a greater number of unique transcripts. RNA was extracted using the PureLink® RNA Mini Kit (Invitrogen, Carlsbad, CA, USA) or using Roche TriPure (Indianapolis, IN, USA), following the manufacturer’s instructions. The grape data set was obtained from NCBI. Details on its generation and record locators can be found in Wen, *et al*.^87^. The grass genomes and annotation were downloaded from Phytozome (phytozome.jgi.doe.gov) and included *Zea mays* 284 5b^+^ ^89^, *Sorghum bicolor* 255 v2.1^90^, *Setaria italica* 312 v2.2^91^, *Oropetium thomaeum* 386 v1.1^92^, *Oryza sativa* 323 v7.0^93^, and *Brachypodium distachyon* 283 v2.1^94^.

### Transcriptome Sequencing

Libraries were prepared using the TruSeq Stranded mRNA Sample Prep Kit (Illumina, Inc., San Diego, CA, USA) or the method described by Wang, *et al*.^95^ (See Supplementary Table S1). Sequencing was performed at the MU DNA Core facility on the campus of the University of Missouri and at Cornell University’s sequencing core facility, and was done on an Illumina Hiseq sequencer with 2 × 100 bp chemistry and six species per lane.

### Sequence Processing

RNA-seq data were quality filtered following standard procedures^96,97^. Transcriptomes were assembled *de novo* using Trinity^98–100^ and processed as described in Yang and Smith (2013).

**Figure 1:**
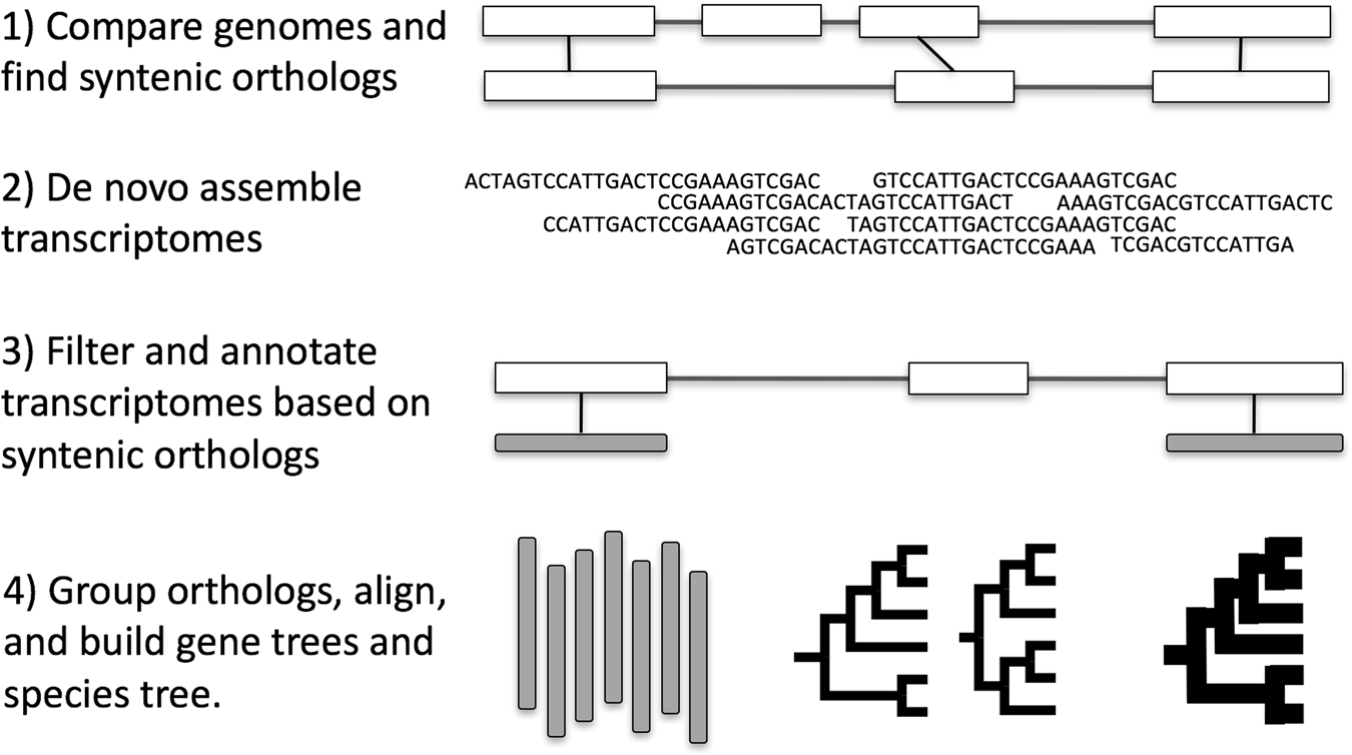
Genome-guided phylo-transcriptomics workflow. Illustration of the workflow followed to produce the genome-guided phylogenies in this study.

The sequenced genomes of *S*. *bicolor* and *S*. *italica* were used for syntenic ortholog determination because both are high quality and publically available, they represent an ingroup and outgroup taxa to the tribe Paniceae, and neither genome contains a recent whole genome duplication event^90,91^. Syntenic orthologs between *S*. *bicolor* and *S*. *italica* were infered using the SynMap tool in CoGe (https://genomevolution.org/CoGe/) with QuotaAlign set to filter out syntenic paralogous regions using a quota setting of 1:1^79,101^. This insures that only genes which are present in a single copy and at the same syntenic location in both species are included. Protein sequences of the *S*. *bicolor* representative of the 1:1 *S*. *italica*/*S*. *bicolor* set were used as the reference sequence for the remainder of the analyses. The assembled tribe Paniceae transcripts (excluding outgroup transcriptomes) were then mapped to the *S*. *bicolor* reference orthologs using BLAST with a cutoff E-value of 0.00001 and 85% amino acid identity. When a given *S*. *bicolor* gene corrisponded to more than one transcript in a species, all transcripts maping to that *S*. *bicolor* gene where discarded to avoid the potential inclussion of paralogs. These sequences where then grouped into orthologous sets for each gene and a multiple alignment was created using mafft^102,103^. In this way, the use of all-by-all BLAST and the MCL algorithm are completely avoided. After further filtering with phyutility and several scripts from Yang and Smith^2^, concatenated trees, coalescence-based quartet summary species trees, and binned coalescence-based quartet summary trees were created using RAxML, ASTRAL, and binning followed by ASTRAL, respectivly^104–107^ (Fig. 1). To investigate syntenic block phylogenies, data from the genome-guided gene trees were grouped based on conserved syntenic blocks across the *S*. *bicolor* and *S*. *italica* genomes (again obtained from CoGe). Each transcript was mapped to its syntenic block and trees created using RAxML based on concatenated transcripts from each syntenic block. The same method was applied to the grape data set, except that the *V*. *vinifera* and *Arabidopsis thaliana* genomes where used and the E-value and protien identity cutoffs where lowered to 0.0001 and 75%, respectively, to account for the increased phylogenetic distances represented in the grape data set. Whole genome duplication events occuring in the ancestor of *A*. *thaliana* but not *V*. *vinifera* are excluded from the analysis because of the 1:1 setting in QuotaAlign. Scripts and instructions for the genome-guided method are available at: bitbucket.org/washjake/transcriptome_phylogeny_tools.

Two gene tree topology-based approaches to orthology inference where also used for comparison: the Agalma pipeline (version 0.5.0) by Dunn, *et al*.^3^ and the Ya Yang pipeline^2,61^. As above, RAxML, ASTRAL, and binning combined with ASTRAL were used to infer phylogenies. Phylogenetic trees and other figures were generated using FigTree, Inkscape, and Vennerable in R^108–111^.

For the grass data used for benchmarking, several additional analyses were run. Single copy syntenic orthologs were found in a pairwise fashion between *O*. *sativa* and each of the other genomes using CoGe as described above. The likelihood of two non-orthologous genes evolving to have not only high sequence similarity, but also to be in the same physical location, and in single-copy across multiple species is incredibly small, giving us high confidence in orthologs inferred using this method. These orthologs where used to create a set of fully synteny-based, one-to-one orthologs across the grasses. While this set does not include all possible single-copy orthologs, it does include all of them for which we can have high confidence based on the available data and current methods, and is hence the best achievable ortholog set for the grasses at the current time. We refer to this as the benchmarking data set.

Each of the ortholog inference methods described above was then run using the transcriptomes generated by the genome sequencing projects referenced above. In this way, the transcripts could be followed by name through the pipelines (except for the Agalma method for which this could not be easily accomplished due to the way the pipeline is packaged). Ortholog sets derived from the genome-guided method and the Yang and Smith method were then compared to the benchmarking set to determine how many orthologs each method was able to find in common with the benchmark orthologs.

### Data Availability

The datasets generated during this study are available in the NCBI SRA repository under the identifiers noted in Supplemental Table S1.

## Results

For species tree inference in the tribe Paniceae, the genome-guided method provided similar numbers of orthologous genes to both the Agalma and Yang & Smith methods at a 90% matrix occupancy cut-off (Table 1). However, for the full matrix runs, when any orthologous gene without all species represented was discarded, the genome-guided method returned fewer orthologs than the other two methods. This is probably due to the genome-guided method not using transcripts that map to the same ortholog. The genome-guided method however, produced more consistent tree topologies than the topology-based methods. For example, all species trees (concatenated, ASTRAL, binned, and with multiple matrix occupancies and taxonomic inclusion) built with the genome-guided orthology pipeline agreed in their subtribe level topologies. The topology-based methods on the other hand, occasionally produced conflicting subtribe-level topologies. In other words, the topology-based methods were more sensitive to perturbations in taxonomic inclusion (when a taxon is removed from the inference to test if the same general topology will result without it) than the genome-guided method. The genome-guided method was also many times faster than the topology-based methods (Table 2).

**Table 1:** Total orthologs found in each method separated by matrix occupancy.

Method			8 spp	90%	Full
Genome-guided	Genes	Total	9,757	2,211	434
		Min	5,389	1,963	434
	Amino Acids	Total	4,182,364	835,229	144,503
		Min	1,775,925	669,215	128,896
Agalma	Genes	Total	11,563	2,308	555
		Min	5,453	2,054	555
	Amino Acids	Total	4,420,707	797,333	182,368
		Min	1,568,329	613,538	168,157
Yang & Smith 1 to 1	Genes	Total	7,323	1,925	898
		Min	3,685	1,781	898
	Amino Acids	Total	2,408,802	789,203	361,901
		Min	1,129,993	628,190	310,283
Yang & Smith MO	Genes	Total	11,568	1,966	1,076
		Min	6,417	1,879	1,076
	Amino Acids	Total	4,362,686	857,857	456,597
		Min	2,009,430	687,942	380,988

**Table 2:** Approximate run times in hours (hrs) for each orthology inference method based on a 16 CPU system.

	Synteny Step	BLAST Step	Alignment and Tree Building for Pruning	Total
Genome-Guided	<1	6.7	N/A	7.7
Agalma	N/A	46.4	88.6	135.0
Yang & Smith	N/A	366.9	412.2	779.1

**Figure 2:**
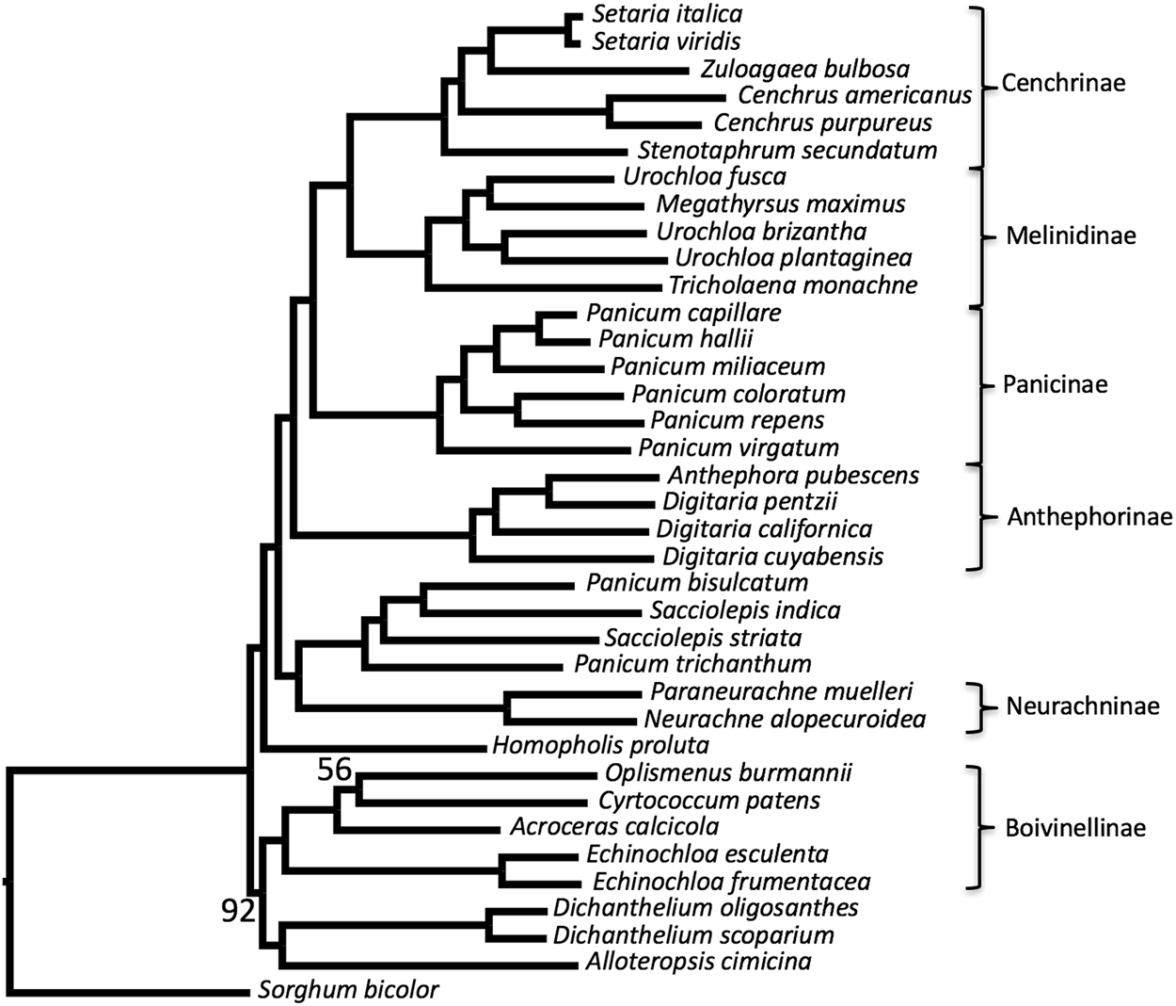
Genome-guided concatenation-based phylogeny of the tribe Paniceae. Phylogenetic tree of the tribe Paniceae (Poaceae) built using RAxML based on a concatenated matrix with 90% gene occupancy. Branches are labeled with maximum likelihood bootstrap values; unlabeled branches have values of 100.

Figure 2 shows what we consider to be the best estimate of the Paniceae nuclear species tree, based on currently available data. This tree places Anthephorinae as direct sister to the MPC clade (subtribes Melinidinae, Panicinae, and Cenchrinae), which, although different from published chloroplast trees^80,82,85^, is consistent with the combined nuclear-chloroplast topology reported by Vicentini, *et al*.^81^.

**Figure 3:**
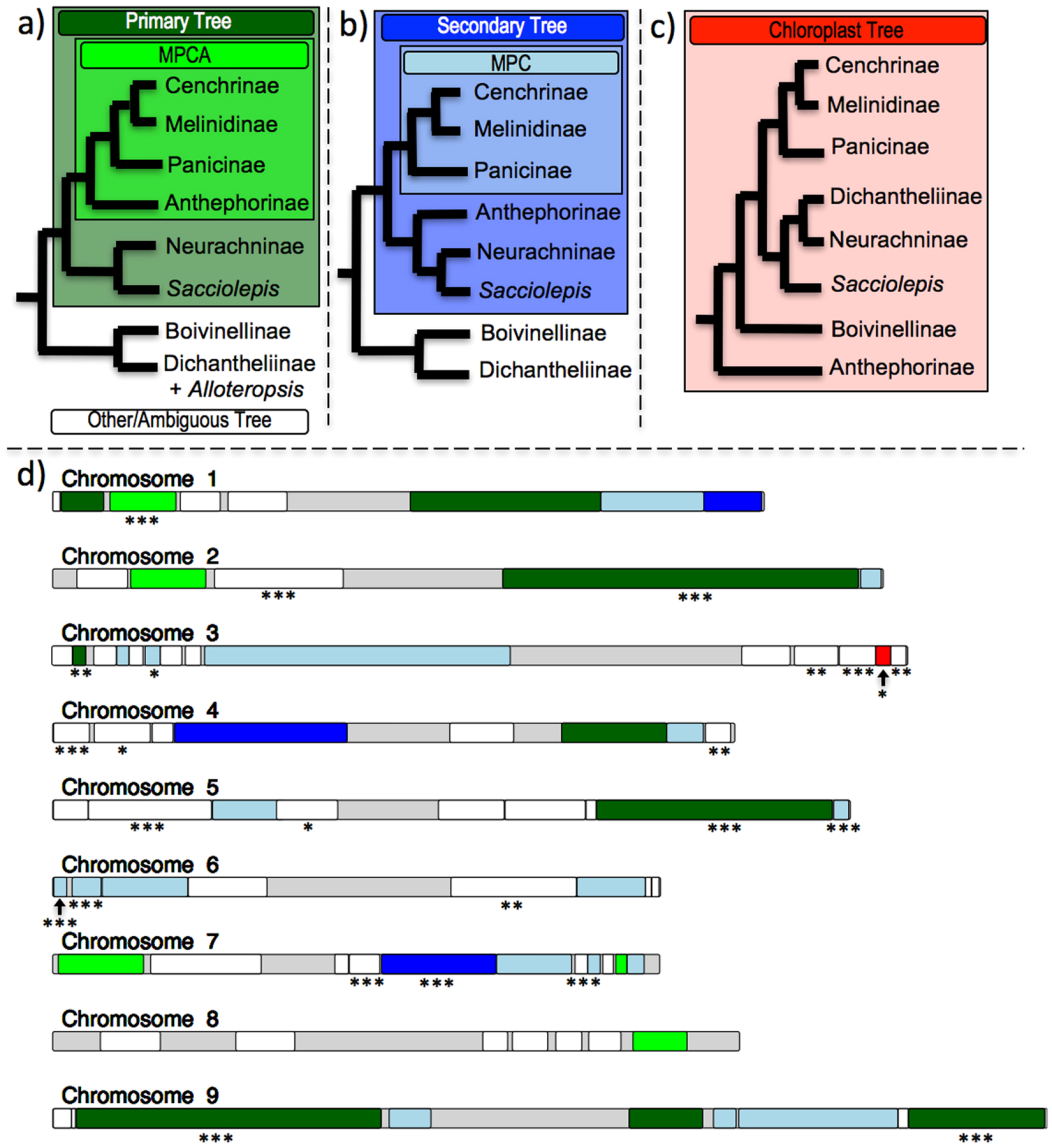
(**a**) Primary nuclear topology found using all methods, (**b**) Secondary nuclear topology, (**c**) Chloroplast topology based on Washburn, *et al*.^80^. (**d**) An ideogram of the *Setaria italica* chromosomes^114^ with conserved syntenic blocks between *S*. *italica* and *Sorghum bicolor* demarcated. Syntenic blocks are colored based on the phylogenetic patterns from a-c that each block supports. Gray indicates areas of the chromosomes not covered by our blocks. Asterisks below the blocks indicate significance level for pairwise Robinson-Foulds distance tests: ***0.001, **0.01, *0.05.

As mentioned, the topology-based approaches (Yang & Smith and Agalma) generally resulted in the same tree topology as the genome-guided method (Fig. 3a). However, in some cases, depending on the taxon sampling included in the analysis, an alternative topology was obtained from these methods. This topology placed the subtribe Anthephorinae together with the Neurachninae and *Sacciolipis* lineages as sister to the MPC clade (Fig. 3b). Internode certainty (IC) scores for the main conflicting node in both the primary and secondary topologies were close to zero and in some cases even negative, suggesting high levels of gene tree incongruence^1,112,113^. Both the genome-guided method, and the topology-based methods included genes representative of each of the *Sorghum bicolor* chromosomes and the major *Setaria italica* scaffolds, indicating that the sampled genes from both methods came from across the entire genome (Supplementary Table S2).

To further dissect the causes of gene tree incongruence within the Paniceae, the tree binning scripts described by Mirarab, *et al*.^106^ where used to separate groups of genes with distinct evolutionary histories. This method allows one to set a bootstrap significance threshold at which branches can be considered high confidence, and then compare large sets of gene trees for compatibility with each other. Different cut-off values were tested for this analysis, almost always (see exception below when a cut-off of 100 was used) resulting in several hundred distinct tree topologies that were incompatible with each other.

When a bootstrap cutoff value of 100 was used as the threshold, indicating that only gene tree branches with 100 percent bootstrap support were considered, the synteny-based data set still placed the trees into 18 unique topology groups (Supplementary Fig. S1). These eighteen topologies were then compared visually and examined for differences that could directly affect the relationship between the MPC clade and the subtribe Anthephorine. Of the eighteen (2211 total genes) topologies, eight topologies (981 total genes) showed strong support for the inclusion of Anthephorine within the MPC (as in the primary topology described above and shown in Figs 2 and 3b), five topologies (615 total genes) showed strong support for Anthephorine as sister to Neurachninae and *Sacciolipis* (as in the secondary topology described above and shown in Fig. 3b), and one topology (123 total genes) agreed with the chloroplast phylogeny from Washburn, *et al*.^80^ (Fig. 3c). The remaining four topologies (492 total genes) had low support for this area of the tree.

Another approach we developed to dissect gene tree incongruence consisted of building trees based on the combination of genes that share a similar physical location. A recent study was able to find likely introgression events using a non-overlapping window approach and constructing trees based on 1 Mb and 100 kb blocks of genes^41^. Because of the genome-guided approach, we were able to group genes into more biologically relevant blocks, namely blocks that are syntenically conserved between ingroup and outgroup taxa. The appearance of a block of genes sharing the same phylogeny, which differs from the species phylogeny, might suggest hybridization/introgression within a group as recently diverged as the Paniceae, but ILS could also produce these types of blocks.

Many syntenic block phylogenies were inconclusive in that they yielded topologies that had little similarity to any of the previously described or published species trees. This seemed to be correlated with the number of genes in a syntenic block in that blocks with more genes generally (but not always) provided a resolved phylogeny that was similar in the placement of the subtribes Melinidinae, Panicinae, Cenchrinae, Anthephorinae, Neurachninae, and the *Sacciolipis* lineage to one of the three phylogenies in Fig. 3. When these blocks and their topologies were mapped to an ideogram^114^ of the *S*. *italica* chromosomes a striking patchwork of differing syntenic block histories was revealed (Fig. 3d). To further investigate whether or not syntenic blocks have distinct tree topologies we used Robinson-Foulds (RF) distances as implemented in the ETE Toolkit^115^. By computing pairwise RF distances for all genes in a given block we created a tree distribution for each of the blocks. We then took the complete set of gene trees (those from all blocks) and randomly re-assigned them to blocks eighty thousand times, each time computing the pairwise RF distance for each block. In this way, we created a simulated “random distribution” of trees for each block that could be used as the null distribution in a statistical test comparing the observed pair-wise RF distances in a block to the simulated distribution under the null hypothesis that all blocks share the same tree distribution. Of the 79 blocks, 15 had distributions that were significantly different than their respective simulated distributions at a significance level of *alpha* <0.001 (Fig. 3d). Hence, it appears that these blocks have a distribution of tree distances smaller than that expected. This observation implies that at least some local regions of the genome have similar evolutionary histories relative to the genome as a whole, either because of locally-coherent ILS or hybridization.

To further benchmark the Genome-Guided method here developed we applied it to two additional data sets. We used publically available sequenced grass genomes to compare our method with the Agalma and Yang and Smith methods. Syntenic relationships between the genomes were used to construct a list of high confidence single copy orthologs across the grasses. This list then served as a benchmark to which the orthologs predicted by each of the methods could be compared. Of course, this list does not contain all orthologs across the grasses, so it cannot tell us anything about the validity of ortholog combinations predicted for genes not found in the list. However, it can tell us when each ortholog prediction method correctly places orthologs in its list, and when it incorrectly identifies paralogs as orthologs. Therefore, we think that these comparisons are informative as to the reliability of different orthology assignment methods.

**Figure 4:**
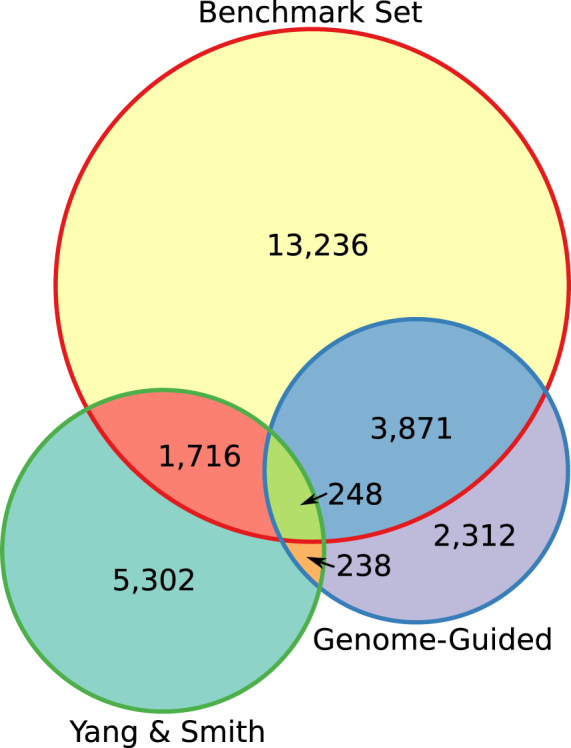
A Venn Diagram comparing the Poaceae gene sets derived from whole genomes, the genome-guided approach, and the Yang & Smith MO pipeline (the 1to1 pipeline is not shown because of large overlap with MO). Diagram created using Inkscape and the R package Vennerable^108,109^.

All three orthology detection pipelines and the synteny-derived benchmarking set generated the same tree topology, in agreement with previous phylogenetic studies, with high confidence^82^. Gene by gene comparisons between each method and the benchmarking set show that the genome-guided method recovers a much higher percentage of ortholog gene trees that agree, in terms of which genes are included, with the benchmarking set than either the Yang & Smith 1 to 1 or MO Methods (62%, 32%, and 31% respectively with a species cutoff of four and genes not found in the benchmarking set excluded. See Table 3 and Fig. 4). Because of the way Agalma is packaged, we were unable to modify its code to include it in this comparison, but it would likely perform similarly to the Yang & Smith method as it uses similar approaches. Beyond the orthologous genes in the benchmarking set, the Yang & Smith methods also include as many as 2,116 additional ortholog gene trees. These trees are based on genes which our direct synteny comparisons did not find (i.e., they did not have a detectable 1:1 orthologous relationship). In some cases, these are genes which the Yang & Smith method mapped in duplicate, for example, two *S*. *italica* genes mapped to one *S*. *bicolor* gene. In other cases, the expected 1:1 syntelog is present for some pairs of species but the *O*. *sativa* syntelogs is not present. These trees may or may not be based on correct orthology assignment, but because they are not in the benchmarking set they could not be evaluated here.

**Table 3:** Grass (Poaceae) wide gene by gene comparisons of orthology detection methods to a benchmark set of orthologs derived entirely from syntenic relationships between sequenced genomes.

Method			4 species	5 species	6 species
Genome-Guided	All Trees Included	Trees Agreeing with Benchmark	4,119	2,169	413
		Total Trees	6,669	3,700	896
		Percent Trees in Agreement	61.8%	58.6%	46.1%
Yang & Smith 1 to 1	All Trees Included	Trees Agreeing with Benchmark	1,936	1,741	1,370
		Total Trees	7,933	6,989	5,171
		Percent Trees in Agreement	24.4%	24.9%	26.5%
	Excluding trees not in benchmark set	Trees Agreeing with Benchmark	1,936	1,741	1,370
		Total Trees	6,088	5,417	4,320
		Percent Trees in Agreement	31.8%	32.1%	31.7%
Yang & Smith MO	All Trees Included	Trees Agreeing with Benchmark	2,000	1,795	1,404
		Total Trees	8,619	7,560	5,464
		Percent Trees in Agreement	23.2%	23.7%	25.7%
	Excluding trees not in benchmark set	Trees Agreeing with Benchmark	2,000	1,795	1,404
		Total Trees	6,503	5,757	4,516
		Percent Trees in Agreement	30.8%	31.2%	31.1%

We also analyzed previously published data from grape and its relatives in order to benchmark the method and explore the phylogenetic distance it is capable of spanning. This dataset was comprised of 17 species spanning from *V*. *vinifera* to *A*. *thaliana*, with the bulk of the species being found within the family Vitaceae (estimated age of ~90 m.y.)^87^. The grape data set performed similarly to the Paniceae dataset we generated in terms of the amount of time it took to perform the genome-guided method versus the other methods. The number of orthologs retrieved was much smaller for the genome-guided method than it was for the two topology-based methods (Supplementary Table S3). This dearth of orthologs is likely due to the simple fact that syntenic relationships are expected to break down as the evolutionary distance between two species increases. Grape and Arabidopsis likely diverged between 69–150 (m.y.) with most estimates around 100 m.y., while Sorghum and Seteria probably diverged between 25–40 m.y.^81,82,88^. Even with substantially fewer genes, the genome-guided method still predicted similar topologies to those of the other two methods and those previously published (Supplementary Fig. S2)^2^.

## Discussion

Phylogenetic consistency, broadly defined as convergence on the “correct” tree topology with increasing data, is a well-established phylogenetic accuracy assessment criterion^116–118^. The Genome-guided method proposed here consistently inferred the same subspecies level tree topology regardless of the matrix occupancy used, the tree building approach applied, and the number of taxa included. The topology-based approaches also resulted in the same tree topology in most cases, however, when the number of taxa were reduced to 33 by removing species near the main areas of conflict (to test the robustness of the topology inference), the topology-based approaches no longer produced consistent results while the Genome-guided method continued to produce the same topology. In general, the inclusion of more taxa, which better represent the diversity of a group of organisms, will increase the accuracy of phylogenetic inference^116,119,120^. It then follows that the topology found by both genome-guided and topology-based methods, when all taxa were included, is likely to be the topological estimate nearest to the true species history. This implies that the genome-guided method should be able to infer that topology with less data than the topology-based methods require for similar confidence and accuracy. Additionally, the grass benchmarking data set comparisons indicate that, of the orthologs we know with high confidence, the genome-guided method predicts a higher percentage of them correctly than does the Yang & Smith method.

Orthology inference with the genome-guided pipeline is also many times faster than the topology-based methods and, except for the CoGe step, can be run efficiently on a standard desktop computer; something not possible with either of topology-based methods. This computational efficiency results from the fact that the genome-guided method does not require all-by-all BLAST or iterative tree pruning steps. The CoGe step is also very simple and straight-forward to run, as is the process of loading new genomes into the CoGe database. CoGe also has the capacity for uploading and analyzing private genomes without making them public and is exceptionally well documented.

A natural downside of the genome-guided method is the need for two genomes that span the taxonomic clade one is working with. While this approach could be used with only one genome or even a list of genes from a *de novo* transcriptome assembly, doing so negates its benefits and will increase the likelihood of including paralogs in the analysis. In these situations, topology-based methods are probably the best analysis choice. Because syntenic relationships often break down with increased phylogenetic distance there is likely a limit to the age of clades for which this method can be applied. Based on our experiments with the grape and Poaceae data sets, the method is functional up to at least 100 m.y. of divergence, and can likely be used successfully in any group for which a reasonable number of orthologs can be found between the ingroup and outgroup taxa. However, our experience indicates that syntenic conservation does not always correlate with divergence time, so the method’s utility will have to be evaluated on a case by case basis.

Based on both the binning analysis and the syntenic block trees, we conclude that the secondary topology, or at least the differential placement of the Anthephorine relative to the MPC, is not an artifact of the topology-based methods, but is supported by an appreciable number of genes regardless of the orthology determination method employed. The different topologies of these genes may result from either ILS or post-speciation hybridization, or both.

The small numbers of transcripts representing many of the syntenic blocks in Fig. 3, likely contributed to an inability to infer well supported phylogenies for some of the blocks. However, RF based topology distribution tests confirmed that tree topology distributions in at least certain areas of the genome are likely more similar for genes in a syntenic block than they are across the whole genome. This type of local-synteny analysis should become even more informative in future studies as more sequenced genomes are generated and included in phylogenetic inference. These types of analyses are also not limited to transcriptomic data but have the potential to add value to other data sets generated with probe/hybridization based data collection methods, as long as one or more sequenced genomes exist within the taxonomic group being studied.

The nuclear phylogeny of the Tribe Paniceae produced in this study is consistent with that produced in a previous study. However, that study was only able to sample one nuclear gene and because the inferred topology was incongruent with the many chloroplast phylogenies of the group, it was generally dismissed. This study demonstrates that in fact the nuclear phylogeny of the Paniceae is very different than the chloroplast one, and that those differences are not due to signals in one or a few genes, but are wide spread across the genome. This study also shows that while that original topology, based on only one nuclear gene is supported by many other genes, not all nuclear genes agree with it, and in fact a significant minority of the genes are incongruent with that topology.

The differences between the nuclear and chloroplast phylogenies shown here are critical to both basic and applied questions within the tribe Paniceae. For example, investigations within the tribe of the evolution of C_4_ photosynthesis, a trait with great economic importance, have focused on the MPC clade at the exclusion of the subtribe Anthephorinae^80,82,121^. Choices about resource investment, such as which genomes to sequence, have also been based almost exclusively on the chloroplast phylogeny^122^. Given our results, further resource investment in Paniceae (at least for the purpose of studying C_4_ photosynthesis) should be directed within the genus *Sacciolepis* or a close relative to it and the subtribe Anthephorinae. We suggest *Sacciolepis indica* as a model C_3_ species for further study as it is a close relative the MPCA clade in both chloroplast and nuclear phylogenies, has a genome size of approximately 523 Mb, and is easily self-pollinated^80^. An ideal Anthephorinae species for further investment is less clear, but *Digitaria cuyabensis* has an approximate genome size of 798 Mb making it a good candidate for genome sequencing^80^. Species within the Crabgrass complex, which includes several different species in the genus *Digitaria*, might also be good candidates for resource investment due to their economic importance as a noxious weed. Data from both nuclear and organellar genes allows for a more informed way to choose future genomes to sequence than simply using organellar data as was done in the past.

## Electronic supplementary material

Supplementary Information
