# Author Correction: Genome-Guided Phylo-Transcriptomic Methods and the Nuclear Phylogenetic Tree of the Paniceae Grasses

**DOI:** 10.1038/s41598-018-25620-4

**Published:** 2018-05-02

**Authors:** Jacob D. Washburn, James C. Schnable, Gavin C. Conant, Thomas P. Brutnell, Ying Shao, Yang Zhang, Martha Ludwig, Gerrit Davidse, J. Chris Pires

**Affiliations:** 1 Division of Biological Sciences, 311 Bond Life Sciences Center, University of Missouri, Columbia, MO 65211 USA; 2 Department of Agronomy & Horticulture, Beadle Center E207, University of Nebraska-Lincoln, Lincoln, NE 68588 USA; 3 Donald Danforth Plant Sciences Center, 975N Warson Rd., St. Louis, MO 63132 USA; 4 Division of Animal Sciences, 920 East Campus Drive, University of Missouri, Columbia, 65211 MO USA; 5 Program in Genetics, Bioinformatics Research Center, Department of Biological Sciences, 356 Ricks Hall, North Carolina State University, Raleigh, NC 27695 USA; 6 St. Jude Children´s Research Hospital, MS 342, Room D-4047E, 262 Danny Thomas Place, Memphis, TN 38105 USA; 7 School of Molecular Sciences, The University of Western Australia (M310), 35 Stirling Highway, Crawley, WA 6009 Australia; 8 Missouri Botanical Garden, P.O. Box 299, St. Louis, Missouri 63166-0299 USA

Correction to: *Scientific Reports* 10.1038/s41598-017-13236-z, published online 19 October 2017

The original version of this Article contained an error in the title of the paper, where the word “Phylogenetic” was incorrectly given as “Phylogentic”. This has now been corrected in the PDF and HTML versions of the Article, and in the accompanying Supplementary Information file.

