# Supplementary material for: Genome-Guided Phylo-Transcriptomic Methods and the Nuclear Phylogenetic Tree of the Paniceae Grasses
Source: Sci Rep. 2017 Oct 19;7:13528. doi: 10.1038/s41598-017-13236-z (PMC5648822; doi:10.1038/s41598-017-13236-z)
Supplement: Supplementary file 1 — Supplementary Information [file 41598_2017_13236_MOESM1_ESM.pdf]

Genome-Guided Phylo-Transcriptomic Methods and the Nuclear Phylogenetic Tree of the Paniceae Grasses

Washburn, Jacob D.*[1]; Schnable, James C.[2,3]; Conant, Gavin C.[4,5]; Brutnell, Thomas P.[3]; Shao, Ying[3,6]; Zhang, Yang[2,3] ; Ludwig, Martha[7]; Davidse, Gerrit[8]; Pires, J. Chris[1]

Supplemental Materials

SUPPLEMENTARY FIGURE S1. Binned concatenated trees.
Trees constructed using a binning cut off value of 100. Branch labels are maximum likelihood bootstrap values.

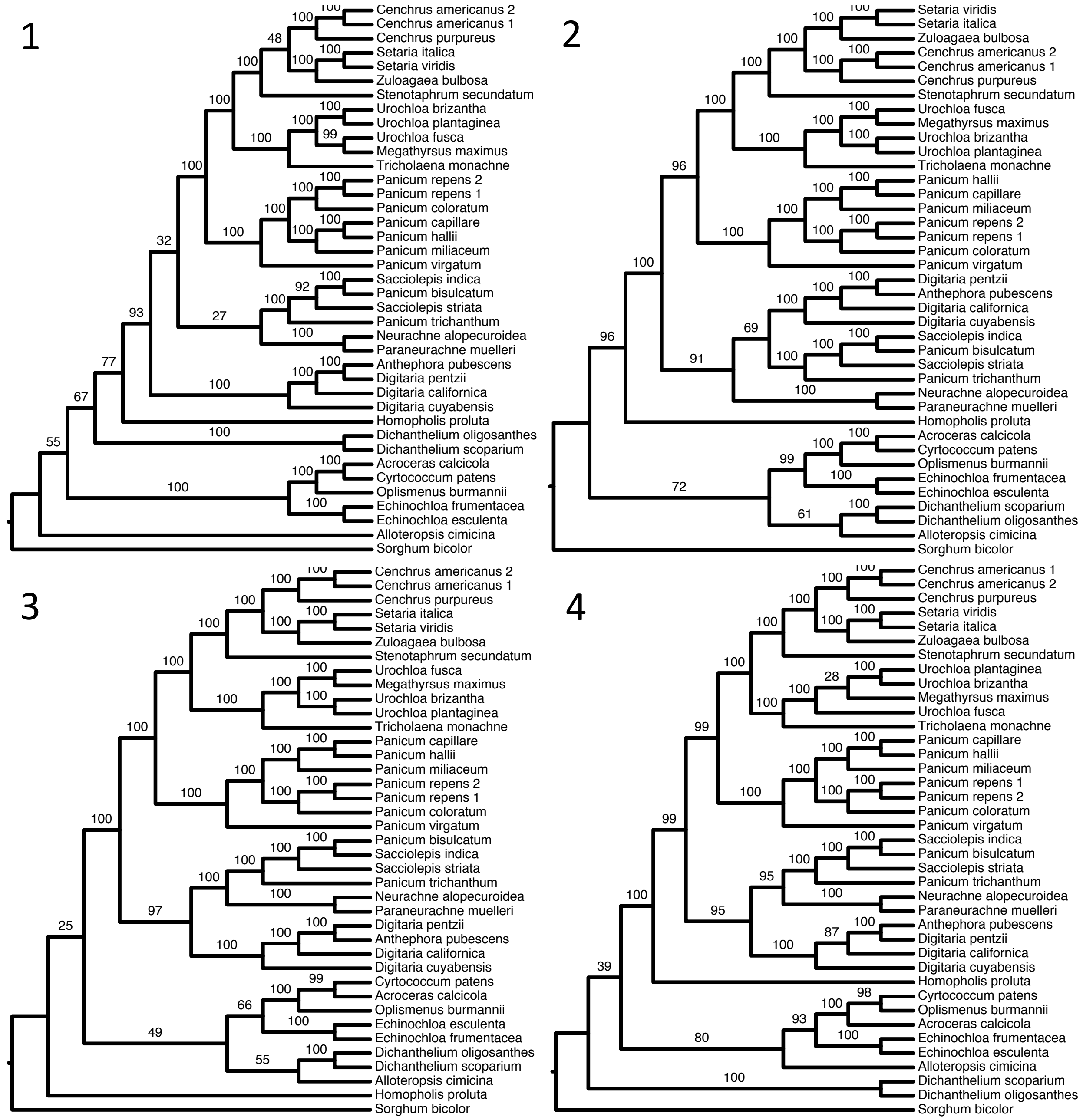

5

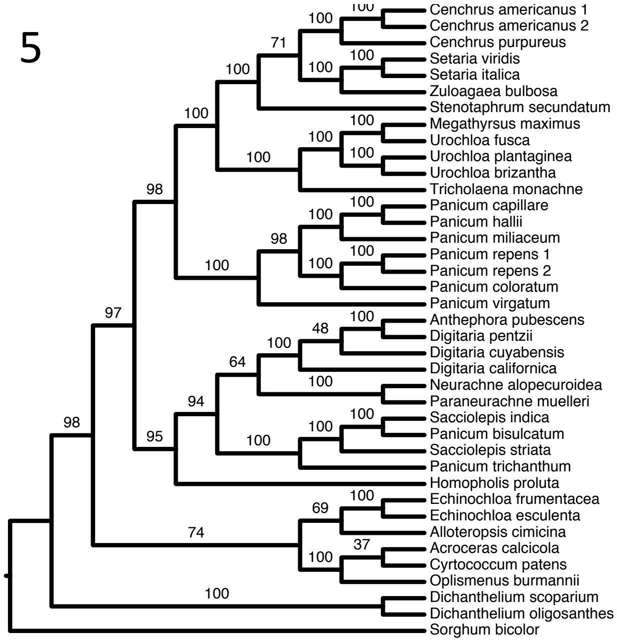
Cenchrus americanus 1
Cenchrus americanus 2
Cenchrus purpureus
Setaria viridis
Setaria italica
Zuloagaea bulbosa
Stenotaphrum secundatum
Megathyrsus maximus
Urochloa fusca
Urochloa plantaginea
Urochloa brizantha
Tricholaena monachne
Panicum capillare
Panicum hallii
Panicum miliaceum
Panicum repens 1
Panicum repens 2
Panicum coloratum
Panicum virgatum
Anthephora pubescens
Digitaria pentzii
Digitaria cuyabensis
Digitaria californica
Neurachne alopecuroidea
Paraneurachne muelleri
Sacciolepis indica
Panicum bisulcatum
Sacciolepis striata
Panicum trichanthum
Homopholis proluta
Echinochloa frumentacea
Echinochloa esculenta
Alloteropsis cimicina
Acroceras calcicola
Cyrtococcum patens
Oplismenus burmannii
Dichanthelium scoparium
Dichanthelium oligosanthes
Sorghum bicolor

6

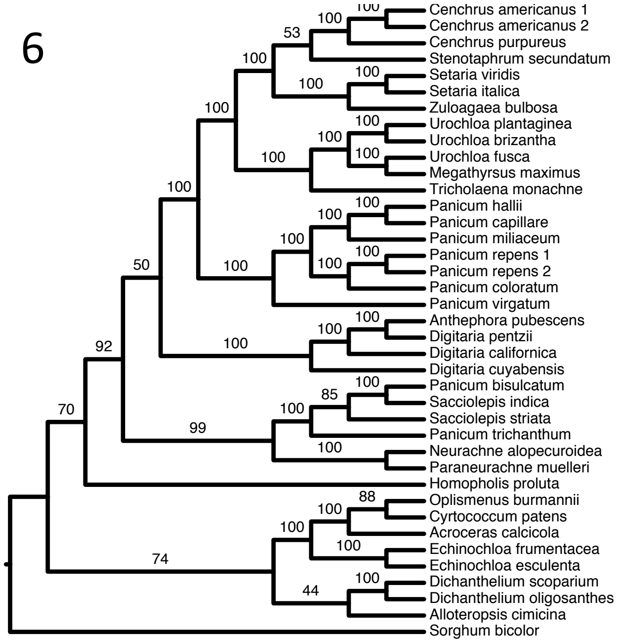
Cenchrus americanus 1
Cenchrus americanus 2
Cenchrus purpureus
Stenotaphrum secundatum
Setaria viridis
Setaria italica
Zuloagaea bulbosa
Urochloa plantaginea
Urochloa brizantha
Urochloa fusca
Megathyrsus maximus
Tricholaena monachne
Panicum hallii
Panicum capillare
Panicum miliaceum
Panicum repens 1
Panicum repens 2
Panicum coloratum
Panicum virgatum
Anthephora pubescens
Digitaria pentzii
Digitaria californica
Digitaria cuyabensis
Panicum bisulcatum
Sacciolepis indica
Sacciolepis striata
Panicum trichanthum
Neurachne alopecuroidea
Paraneurachne muelleri
Homopholis proluta
Oplismenus burmannii
Cyrtococcum patens
Acroceras calcicola
Echinochloa frumentacea
Echinochloa esculenta
Dichanthelium scoparium
Dichanthelium oligosanthes
Alloteropsis cimicina
Sorghum bicolor

7

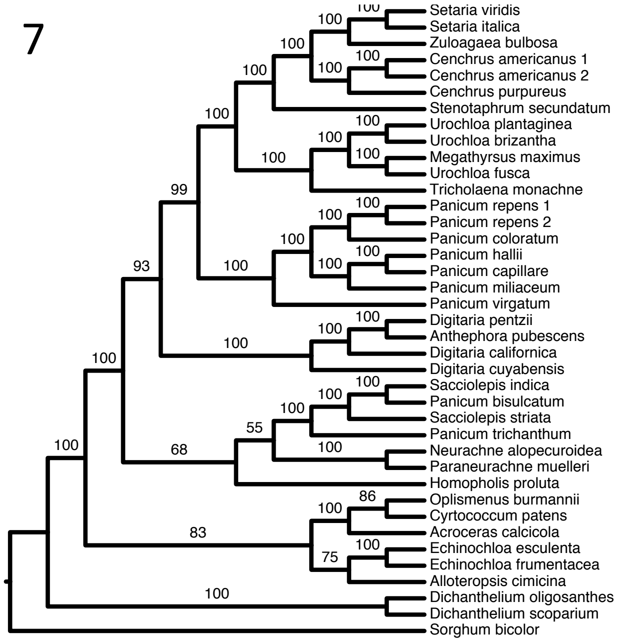
Setaria viridis
Setaria italica
Zuloagaea bulbosa
Cenchrus americanus 1
Cenchrus americanus 2
Cenchrus purpureus
Stenotaphrum secundatum
Urochloa plantaginea
Urochloa brizantha
Megathyrsus maximus
Urochloa fusca
Tricholaena monachne
Panicum repens 1
Panicum repens 2
Panicum coloratum
Panicum hallii
Panicum capillare
Panicum miliaceum
Panicum virgatum
Digitaria pentzii
Anthephora pubescens
Digitaria californica
Digitaria cuyabensis
Sacciolepis indica
Panicum bisulcatum
Sacciolepis striata
Panicum trichanthum
Neurachne alopecuroidea
Paraneurachne muelleri
Homopholis proluta
Oplismenus burmannii
Cyrtococcum patens
Acroceras calcicola
Echinochloa esculenta
Echinochloa frumentacea
Alloteropsis cimicina
Dichanthelium oligosanthes
Dichanthelium scoparium
Sorghum bicolor

8

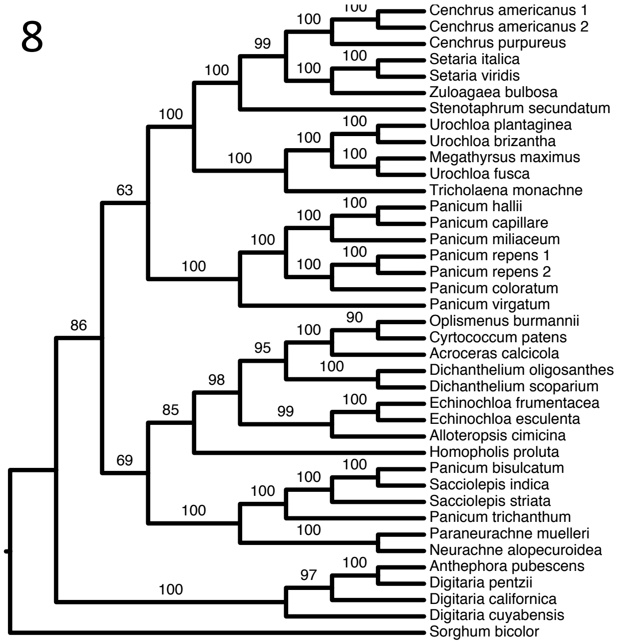
Cenchrus americanus 1
Cenchrus americanus 2
Cenchrus purpureus
Setaria italica
Setaria viridis
Zuloagaea bulbosa
Stenotaphrum secundatum
Urochloa plantaginea
Urochloa brizantha
Megathyrsus maximus
Urochloa fusca
Tricholaena monachne
Panicum hallii
Panicum capillare
Panicum miliaceum
Panicum repens 1
Panicum repens 2
Panicum coloratum
Panicum virgatum
Oplismenus burmannii
Cyrtococcum patens
Acroceras calcicola
Dichanthelium oligosanthes
Dichanthelium scoparium
Echinochloa frumentacea
Echinochloa esculenta
Alloteropsis cimicina
Homopholis proluta
Panicum bisulcatum
Sacciolepis indica
Sacciolepis striata
Panicum trichanthum
Paraneurachne muelleri
Neurachne alopecuroidea
Anthephora pubescens
Digitaria pentzii
Digitaria californica
Digitaria cuyabensis
Sorghum bicolor

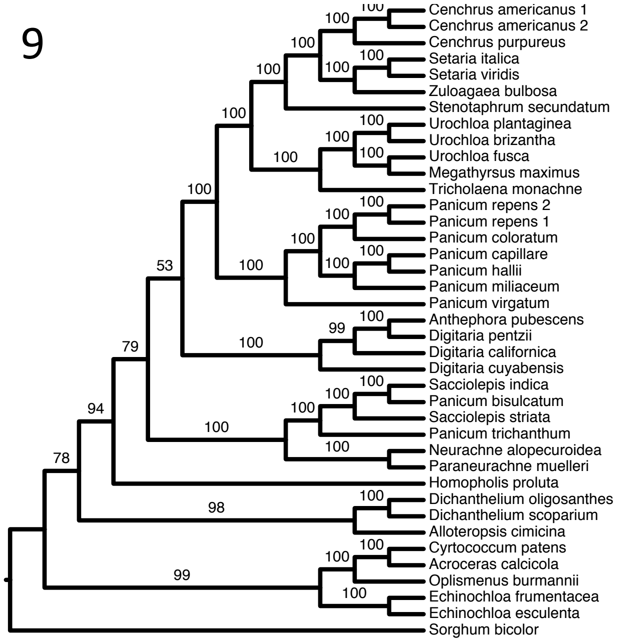
9
Cenchrus americanus 1
Cenchrus americanus 2
Cenchrus purpureus
Setaria italica
Setaria viridis
Zuloagaea bulbosa
Stenotaphrum secundatum
Urochloa plantaginea
Urochloa brizantha
Urochloa fusca
Megathyrsus maximus
Tricholaena monachne
Panicum repens 2
Panicum repens 1
Panicum coloratum
Panicum capillare
Panicum hallii
Panicum miliaceum
Panicum virgatum
Anthephora pubescens
Digitaria pentzii
Digitaria californica
Digitaria cuyabensis
Sacciolepis indica
Panicum bisulcatum
Sacciolepis striata
Panicum trichanthum
Neurachne alopecuroidea
Paraneurachne muelleri
Homopholis proluta
Dichanthelium oligosanthes
Dichanthelium scoparium
Alloteropsis cimicina
Cyrtococcum patens
Acroceras calcicola
Oplismenus burmannii
Echinochloa frumentacea
Echinochloa esculenta
Sorghum bicolor

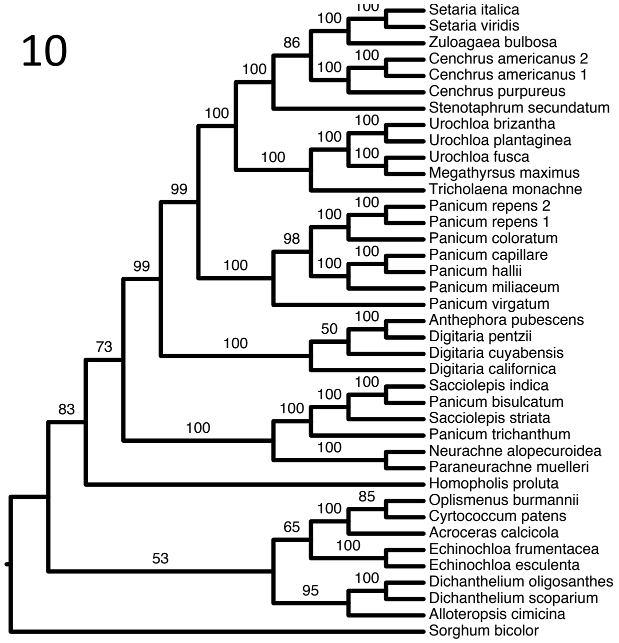
10
Setaria italica
Setaria viridis
Zuloagaea bulbosa
Cenchrus americanus 2
Cenchrus americanus 1
Cenchrus purpureus
Stenotaphrum secundatum
Urochloa brizantha
Urochloa plantaginea
Urochloa fusca
Megathyrsus maximus
Tricholaena monachne
Panicum repens 2
Panicum repens 1
Panicum coloratum
Panicum capillare
Panicum hallii
Panicum miliaceum
Panicum virgatum
Anthephora pubescens
Digitaria pentzii
Digitaria cuyabensis
Digitaria californica
Sacciolepis indica
Panicum bisulcatum
Sacciolepis striata
Panicum trichanthum
Neurachne alopecuroidea
Paraneurachne muelleri
Homopholis proluta
Oplismenus burmannii
Cyrtococcum patens
Acroceras calcicola
Echinochloa frumentacea
Echinochloa esculenta
Dichanthelium oligosanthes
Dichanthelium scoparium
Alloteropsis cimicina
Sorghum bicolor

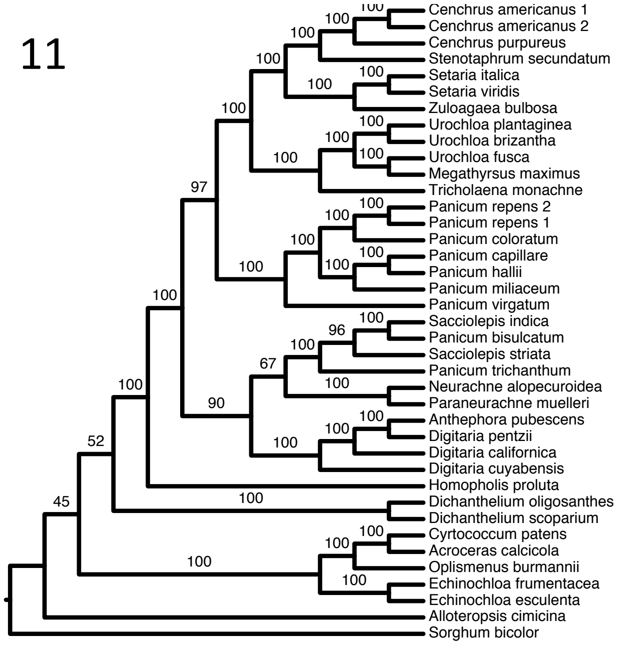
11
Cenchrus americanus 1
Cenchrus americanus 2
Cenchrus purpureus
Stenotaphrum secundatum
Setaria italica
Setaria viridis
Zuloagaea bulbosa
Urochloa plantaginea
Urochloa brizantha
Urochloa fusca
Megathyrsus maximus
Tricholaena monachne
Panicum repens 2
Panicum repens 1
Panicum coloratum
Panicum capillare
Panicum hallii
Panicum miliaceum
Panicum virgatum
Sacciolepis indica
Panicum bisulcatum
Sacciolepis striata
Panicum trichanthum
Neurachne alopecuroidea
Paraneurachne muelleri
Anthephora pubescens
Digitaria pentzii
Digitaria californica
Digitaria cuyabensis
Homopholis proluta
Dichanthelium oligosanthes
Dichanthelium scoparium
Cyrtococcum patens
Acroceras calcicola
Oplismenus burmannii
Echinochloa frumentacea
Echinochloa esculenta
Alloteropsis cimicina
Sorghum bicolor

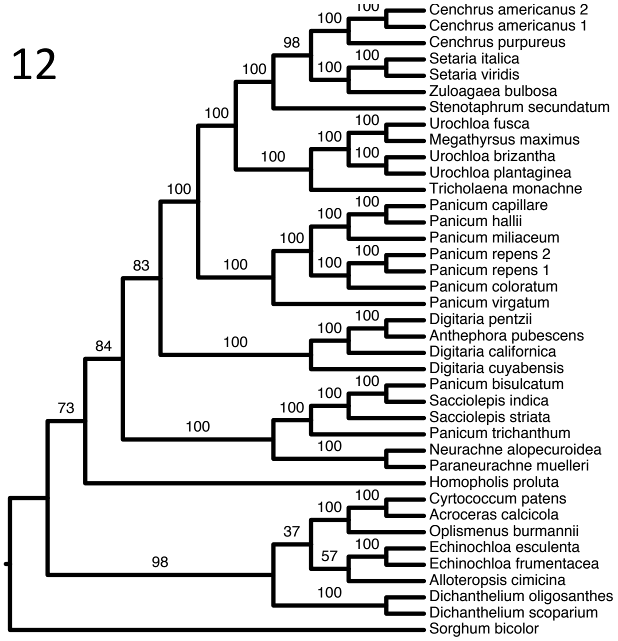
12
Cenchrus americanus 2
Cenchrus americanus 1
Cenchrus purpureus
Setaria italica
Setaria viridis
Zuloagaea bulbosa
Stenotaphrum secundatum
Urochloa fusca
Megathyrsus maximus
Urochloa brizantha
Urochloa plantaginea
Tricholaena monachne
Panicum capillare
Panicum hallii
Panicum miliaceum
Panicum repens 2
Panicum repens 1
Panicum coloratum
Panicum virgatum
Digitaria pentzii
Anthephora pubescens
Digitaria californica
Digitaria cuyabensis
Panicum bisulcatum
Sacciolepis indica
Sacciolepis striata
Panicum trichanthum
Neurachne alopecuroidea
Paraneurachne muelleri
Homopholis proluta
Cyrtococcum patens
Acroceras calcicola
Oplismenus burmannii
Echinochloa esculenta
Echinochloa frumentacea
Alloteropsis cimicina
Dichanthelium oligosanthes
Dichanthelium scoparium
Sorghum bicolor

13

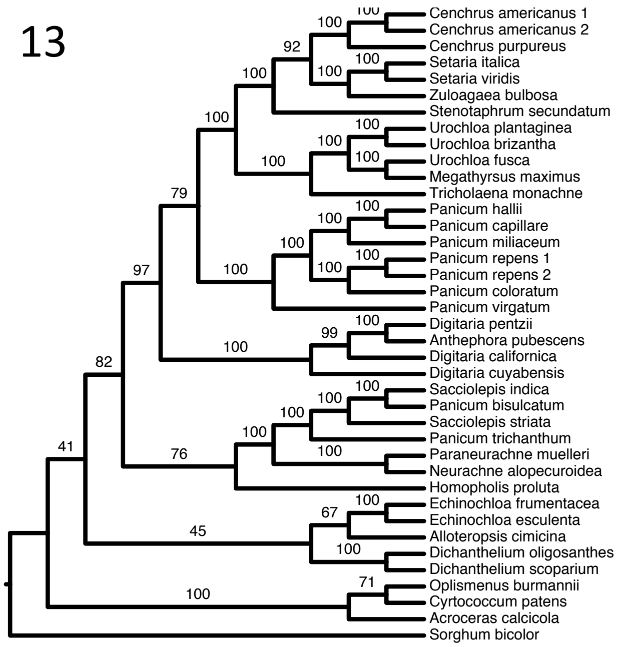
Cenchrus americanus 1
Cenchrus americanus 2
Cenchrus purpureus
Setaria italica
Setaria viridis
Zuloagaea bulbosa
Stenotaphrum secundatum
Urochloa plantaginea
Urochloa brizantha
Urochloa fusca
Megathyrsus maximus
Tricholaena monachne
Panicum hallii
Panicum capillare
Panicum miliaceum
Panicum repens 1
Panicum repens 2
Panicum coloratum
Panicum virgatum
Digitaria pentzii
Anthephora pubescens
Digitaria californica
Digitaria cuyabensis
Sacciolepis indica
Panicum bisulcatum
Sacciolepis striata
Panicum trichanthum
Paraneurachne muelleri
Neurachne alopecuroidea
Homopholis proluta
Echinochloa frumentacea
Echinochloa esculenta
Alloteropsis cimicina
Dichanthelium oligosanthes
Dichanthelium scoparium
Oplismenus burmannii
Cyrtococcum patens
Acroceras calcicola
Sorghum bicolor

14

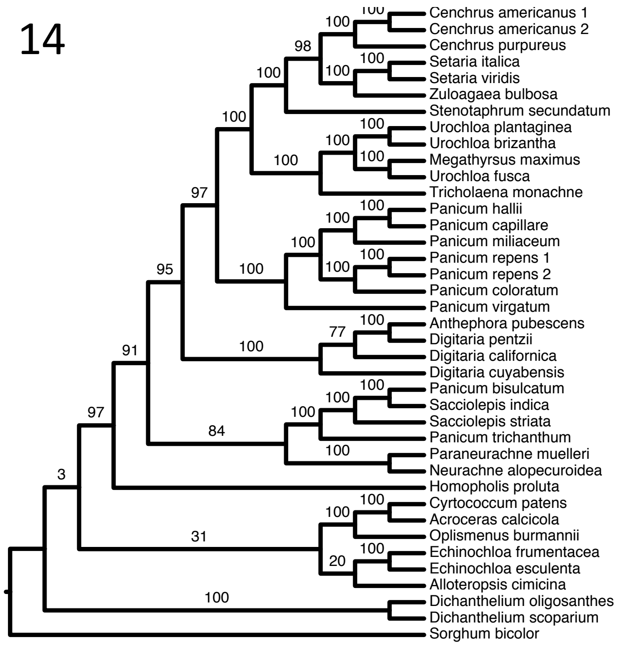
Cenchrus americanus 1
Cenchrus americanus 2
Cenchrus purpureus
Setaria italica
Setaria viridis
Zuloagaea bulbosa
Stenotaphrum secundatum
Urochloa plantaginea
Urochloa brizantha
Megathyrsus maximus
Urochloa fusca
Tricholaena monachne
Panicum hallii
Panicum capillare
Panicum miliaceum
Panicum repens 1
Panicum repens 2
Panicum coloratum
Panicum virgatum
Anthephora pubescens
Digitaria pentzii
Digitaria californica
Digitaria cuyabensis
Panicum bisulcatum
Sacciolepis indica
Sacciolepis striata
Panicum trichanthum
Paraneurachne muelleri
Neurachne alopecuroidea
Homopholis proluta
Cyrtococcum patens
Acroceras calcicola
Oplismenus burmannii
Echinochloa frumentacea
Echinochloa esculenta
Alloteropsis cimicina
Dichanthelium oligosanthes
Dichanthelium scoparium
Sorghum bicolor

15

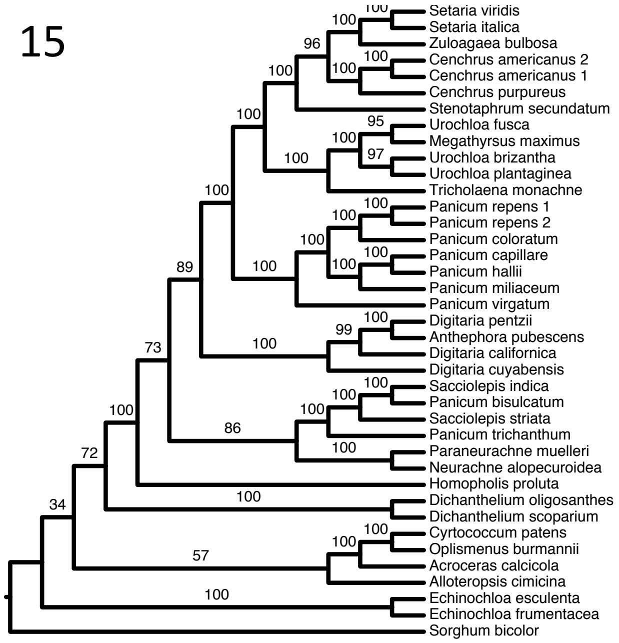
Setaria viridis
Setaria italica
Zuloagaea bulbosa
Cenchrus americanus 2
Cenchrus americanus 1
Cenchrus purpureus
Stenotaphrum secundatum
Urochloa fusca
Megathyrsus maximus
Urochloa brizantha
Urochloa plantaginea
Tricholaena monachne
Panicum repens 1
Panicum repens 2
Panicum coloratum
Panicum capillare
Panicum hallii
Panicum miliaceum
Panicum virgatum
Digitaria pentzii
Anthephora pubescens
Digitaria californica
Digitaria cuyabensis
Sacciolepis indica
Panicum bisulcatum
Sacciolepis striata
Panicum trichanthum
Paraneurachne muelleri
Neurachne alopecuroidea
Homopholis proluta
Dichanthelium oligosanthes
Dichanthelium scoparium
Cyrtococcum patens
Oplismenus burmannii
Acroceras calcicola
Alloteropsis cimicina
Echinochloa esculenta
Echinochloa frumentacea
Sorghum bicolor

16

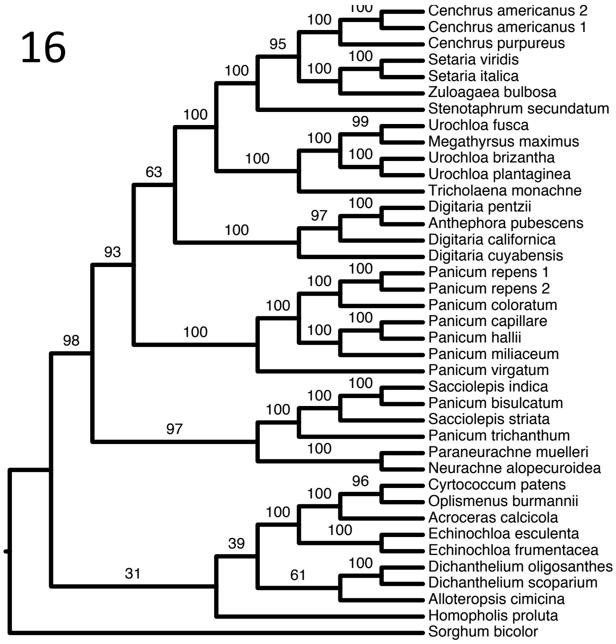
Cenchrus americanus 2
Cenchrus americanus 1
Cenchrus purpureus
Setaria viridis
Setaria italica
Zuloagaea bulbosa
Stenotaphrum secundatum
Urochloa fusca
Megathyrsus maximus
Urochloa brizantha
Urochloa plantaginea
Tricholaena monachne
Digitaria pentzii
Anthephora pubescens
Digitaria californica
Digitaria cuyabensis
Panicum repens 1
Panicum repens 2
Panicum coloratum
Panicum capillare
Panicum hallii
Panicum miliaceum
Panicum virgatum
Sacciolepis indica
Panicum bisulcatum
Sacciolepis striata
Panicum trichanthum
Paraneurachne muelleri
Neurachne alopecuroidea
Cyrtococcum patens
Oplismenus burmannii
Acroceras calcicola
Echinochloa esculenta
Echinochloa frumentacea
Dichanthelium oligosanthes
Dichanthelium scoparium
Alloteropsis cimicina
Homopholis proluta
Sorghum bicolor

17

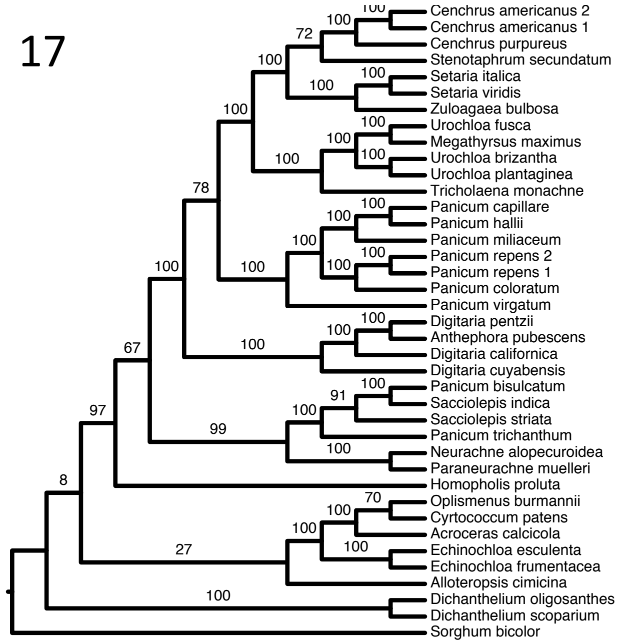
Cenchrus americanus 2
Cenchrus americanus 1
Cenchrus purpureus
Stenotaphrum secundatum
Setaria italica
Setaria viridis
Zuloagaea bulbosa
Urochloa fusca
Megathyrsus maximus
Urochloa brizantha
Urochloa plantaginea
Tricholaena monachne
Panicum capillare
Panicum hallii
Panicum miliaceum
Panicum repens 2
Panicum repens 1
Panicum coloratum
Panicum virgatum
Digitaria pentzii
Anthephora pubescens
Digitaria californica
Digitaria cuyabensis
Panicum bisulcatum
Sacciolepis indica
Sacciolepis striata
Panicum trichanthum
Neurachne alopecuroidea
Paraneurachne muelleri
Homopholis proluta
Oplismenus burmannii
Cyrtococcum patens
Acroceras calcicola
Echinochloa esculenta
Echinochloa frumentacea
Alloteropsis cimicina
Dichanthelium oligosanthes
Dichanthelium scoparium
Sorghum bicolor

18

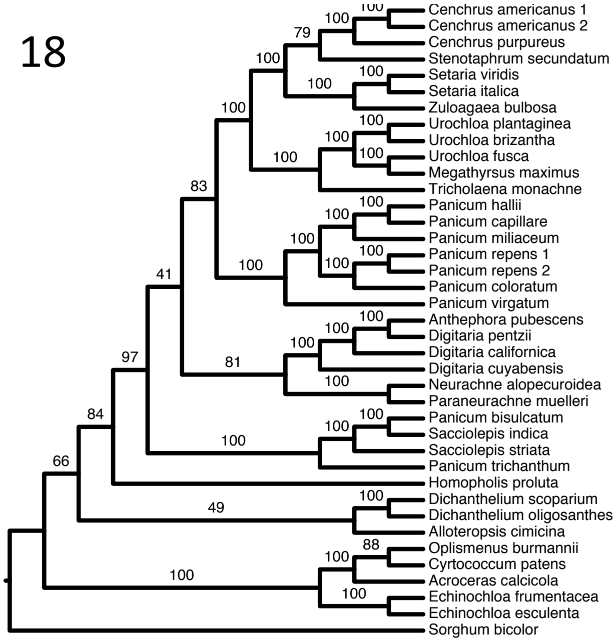
Cenchrus americanus 1
Cenchrus americanus 2
Cenchrus purpureus
Stenotaphrum secundatum
Setaria viridis
Setaria italica
Zuloagaea bulbosa
Urochloa plantaginea
Urochloa brizantha
Urochloa fusca
Megathyrsus maximus
Tricholaena monachne
Panicum hallii
Panicum capillare
Panicum miliaceum
Panicum repens 1
Panicum repens 2
Panicum coloratum
Panicum virgatum
Anthephora pubescens
Digitaria pentzii
Digitaria californica
Digitaria cuyabensis
Neurachne alopecuroidea
Paraneurachne muelleri
Panicum bisulcatum
Sacciolepis indica
Sacciolepis striata
Panicum trichanthum
Homopholis proluta
Dichanthelium scoparium
Dichanthelium oligosanthes
Alloteropsis cimicina
Oplismenus burmannii
Cyrtococcum patens
Acroceras calcicola
Echinochloa frumentacea
Echinochloa esculenta
Sorghum bicolor

SUPPLEMENTARY FIGURE S2. Tree built using the Grape data based on the genome-guided method. Trees from all three methods shared this same topology.

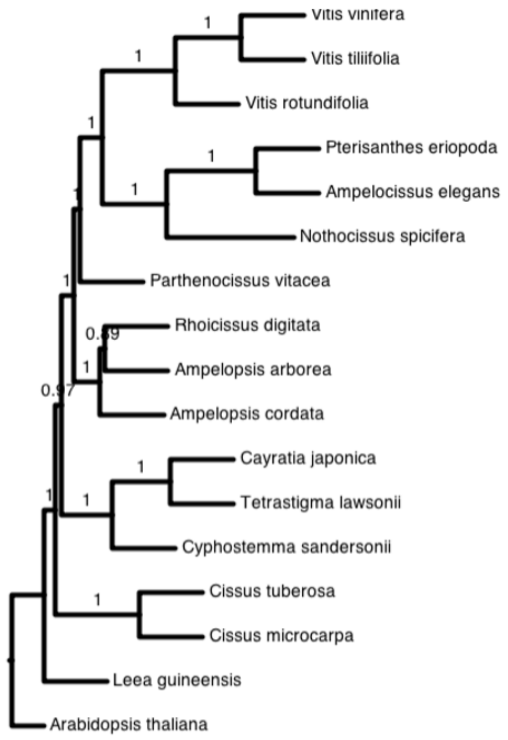

SUPPLEMENTARY TABLE S1. Materials used in study.
A list of all plant species used in the study along with their source, identification number, herbarium specimen number, and NCBI record numbers where applicable.

Subtribe	Genus	Species	Authority	Source	ID number	Herbarium Accession No.	NCBI Number	Library Method
Cenchrinae	*Cenchrus*	*americanus*	(L.) Morrone	KD	ICMP-451	MO-6635001	SRR5406199	TS
Cenchrinae	*Cenchrus*	*americanus*	(L.) Morrone	USDA	PI 279664	N/A	SRR5406198	W
Cenchrinae	*Cenchrus*	*purpureus*	(Schumach.) Morrone	USDA	PI 667860	N/A	SRR5406197	TS
Cenchrinae	*Setaria*	*viridis*	(L.) P. Beauv.	N/A	N/A	N/A	ERR385861-6	N/A
Cenchrinae	*Stenotaphrum*	*secundatum*	(Walter) Kuntze	USDA	PI 410357	MO-6635005	SRR5406196	TS
Cenchrinae	*Zuloagaea*	*bulbosa*	(Kunth) Bess	USDA	PI 442528	MO-6635006	SRR5406195	TS
Melinidinae	*Megathyrsus*	*maximus*	(Jacq.) B.K. Simon & S.W.L. Jacobs	USDA	PI 404634	MO-6635008	SRR5406194	TS
Melinidinae	*Tricholaena*	*monachne*	(Trin.) Stapf & C.E. Hubb.	USDA	PI 166381	MO-6635009	SRR5406193	TS
Melinidinae	*Urochloa*	*brizantha*	(Hochst. ex A. Rich.) R. Webster	USDA	PI 226049	MO-6635010	SRR5406192	W
Melinidinae	*Urochloa*	*fusca*	(Sw.) B.F. Hansen & Wunderlin	USDA	LBJWC-52	MO-6635011	SRR5406191	W
Melinidinae	*Urochloa*	*plantaginea*	(Link) R.D. Webster	USDA	PI 379628	MO-6635012	SRR5406190	TS
Panicinae	*Panicum*	*capillare*	L.	USDA	PI 220025	MO-6635013	SRR5406189	TS
Panicinae	*Panicum*	*coloratum*	L.	USDA	PI 185546	MO-6635014	SRR5406188	TS
Panicinae	*Panicum*	*hallii*	Vasey	DL	HAL 2	MO-6635015	SRR5406187	TS
Panicinae	*Panicum*	*miliaceum*	L.	USDA	PI 578073	MO-6635016	SRR5406186	W
Panicinae	*Panicum*	*repens*	L.	USDA	PI 208687	MO-6635017	SRR5406185	TS
Panicinae	*Panicum*	*repens*	L.	USDA	PI 238344	MO-6635018	SRR5406184	TS
Panicinae	*Panicum*	*virgatum*	L.	LEB	AP13	MO-6635019	SRR5406183	TS
Dichantheliinae	*Dichanthelium*	*oligosanthes*	(Schult.) Gould	AS	D1	MO-6635020	SRR5406182	W
Dichantheliinae	*Dichanthelium*	*scoparium*	(Lam.) Gould	USDA	PI 652864	MO-6635021	SRR5406181	TS
Incertae sedis	*Panicum*	*bisulcatum*	Thunb.	USDA	PI 286485	MO-6647157	SRR5406180	TS
Incertae sedis	*Panicum*	*trichanthum*	Nees	USDA	PI 206329	MO-6647158	SRR5406179	TS
Incertae sedis	*Sacciolepis*	*indica*	(L.) Chase	USDA	PI 338609	MO-6635022	SRR5406178	TS
Incertae sedis	*Sacciolepis*	*striata*	(L.) Nash	USDA	NSL 454620	MO-6635023	SRR5406177	TS
Incertae sedis	*Walwhalleya*	*proluta*	(F. Muell.) K. E. Wills & J. J. Bruhl	NS	NS 42146	MO-6647159	SRR5406176	TS
Neurachninae	*Neurachne*	*alopecuroidea*	R. Br.	ML	N/A	N/A	SRR5406175	TS
Neurachninae	*Paraneurachne*	*muelleri*	(Hack.) S.T.Blake	ML	N/A	N/A	SRR5406174	TS
Boivinellinae	*Acroceras*	*calcicola*	A. Camus	MSB	MSB 199378	MO-6647161	SRR5406173	TS
Boivinellinae	*Alloteropsis*	*cimicina*	(L.) Stapf	JRB	JRB	MO-6635025	SRR5406172	TS
Boivinellinae	*Cyrtococcum*	*patens*	(L.) A. Camus	MSB	MSB 516	MO-6647160	SRR5406171	TS
Boivinellinae	*Echinochloa*	*esculenta*	(A. Braun) H. Scholz	USDA	PI 647850	MO-6635026	SRR5406170	W
Boivinellinae	*Echinochloa*	*frumentacea*	Link	USDA	Ames 11429	MO-6635027	SRR5406169	W
Boivinellinae	*Oplismenus*	*burmannii*	(Retz.) P. Beauv.	MW	MW	MO-6635028	SRR5406168	TS
Anthephorinae	*Anthephora*	*pubescens*	Nees	EK	TK1	MO-6635029	SRR5406167	W
Anthephorinae	*Digitaria*	*californica*	(Benth.) Henrard	USDA	PI 364670	MO-6635030	SRR5406166	TS
Anthephorinae	*Digitaria*	*cuyabensis*	(Trin.) Parodi	USDA	PI 349688	MO-6635031	SRR5406165	TS
Anthephorinae	*Digitaria*	*pentzii*	Stent	USDA	PI 476678	MO-6635032	SRR5406164	TS
Sorghinae	*Dichanthium*	*sericeum*	(R. Br.) A. Camus	USDA	PI 213880	MO-6635033	SRR5406163	TS
Arundinelleae	*Arundinella*	*hirta*	(Thunb.) Tanaka	USDA	PI 246756	MO-6647156	SRR5406162	W
Arundinelleae	*Arundinella*	*hookeri*	Munro ex Keng	EK	Kew #0050290	N/A	SRR5406161	W
Paspalinae	*Paspalum*	*vaginatum*	Sw.	USDA	PI 509022	MO-6635035	SRR5406160	W
Otachyriinae	*Steinchisma*	*decipiens*	(Nees ex Trin.) W. V. Br.	USDA	PI 462236	MO-6635036	SRR5406159	TS
Arthropogoinae	*Coleateania*	*prionitis*	(Nees) Soreng	USDA	PI 496395	MO-6635037	SRR5406158	W
Tristachyideae	*Danthoniopsis*	*dinteri*	(Pilg.) C.E. Hubb.	USDA	PI 207548	MO-6635038	SRR5406157	W
	Eriachne	*aristidea*	F. Muell.	USDA	PI 238306	MO-6635039	SRR5406156	W
	Aristida	*congesta*	Roem. & Schult.	USDA	PI 364389	MO-6635040	SRR5406155	W
	Aristida	*purpurea*	Nutt.	USDA	PI 598972	N/A	SRR5406154	W

Source abreviations: MSB=Millenial Seed Bank, NS=Nindethana Australian Seeds, KD=K.M. Devos, DL=D.B. Lowery, LEB=L.E. Bartley, AS=A.J. Studer, ML= M. Ludwig, JRB=J.R. Burkhalter, MW=W.M. Whitten, EK=E.A. Kellogg. Library methods: TS=TruSeq Stranded mRNA, W=Wang, et al. (2011).

SUPPLEMENTARY TABLE S2. Total orthologs found on each *Sorghum bicolor* and *Setaria italica* chromosome separated by matrix occupancy and orthology inference method.

		Original	Genome-guided			Agalma			Yang & Smith 1 to 1			Yang & Smith MO		
			8_spp	90%	Full	8_spp	90%	Full	8_spp	90%	Full	8_spp	90%	Full
		Genes	Genes	Genes	Genes	Genes	Genes	Genes	Genes	Genes	Genes	Genes	Genes	Genes
S.bicolor Chromosomes	1	3,289	2,018	511	103	1,240	398	106	871	368	167	1,399	375	198
	2	2,181	1,249	293	77	749	257	80	511	201	108	817	210	127
	3	2,587	1,498	335	61	885	275	67	583	235	114	979	241	135
	4	2,112	1,264	297	61	778	250	61	491	198	89	823	193	106
	5	681	294	50	6	183	43	7	103	39	14	200	40	17
	6	1,558	876	177	32	558	166	50	361	128	68	604	139	85
	7	1,073	583	132	19	361	117	22	210	96	45	365	98	53
	8	753	375	78	12	239	76	15	125	48	23	234	58	30
	9	1,434	828	160	25	461	136	30	314	127	58	476	113	69
	10	1,365	751	174	38	456	118	33	284	110	54	470	115	66
Total*		17,033	9,736	2,207	434	5,910	1,836	471	3,853	1,550	740	6,367	1,582	886
S. italica Scaffolds	1	2,112	1,264	297	61	778	250	61	491	198	89	823	193	106
	2	2,172	1,246	294	76	748	255	80	505	198	107	813	209	127
	3	2,091	1,191	246	39	710	219	58	474	192	95	744	188	115
	4	1,365	751	174	38	456	118	33	284	110	54	470	115	66
	5	2,587	1,498	335	61	885	275	67	583	235	114	979	241	135
	6	1,073	583	132	19	361	117	22	210	96	45	365	98	53
	7	1,633	881	172	30	541	156	37	323	113	53	559	121	66
	8	690	295	47	6	186	45	7	103	37	15	204	40	19
	9	3,298	2,023	512	103	1,244	398	106	872	368	167	1,400	375	198
Total*		17,021	9,732	2,209	433	5,909	1,833	471	3,845	1,547	739	6,357	1,580	885

* Differences in totals due to incomplete genome assemblies

SUPPLEMENTARY TABLE S3. Total orthologs found in each method for the Grape data set.

Genome-guided				Agalma				Yang & Smith 1 to 1				Yang & Smith MO			
Genes		Amino Acids		Genes		Amino Acids		Genes		Amino Acids		Genes		Amino Acids	
Total	Min	Total	Min	Total	Min	Total	Min	Total	Min	Total	Min	Total	Min	Total	Min
1,677	437	642,010	112,568	12,744	5,151	4,608,990	1,686,999	13,342	3,939	3,926,469	1,317,706	17,181	6,796	5,871,359	2,713,463
